# On the Prediction of Biogas Production from Vegetables, Fruits, and Food Wastes by ANFIS- and LSSVM-Based Models

**DOI:** 10.1155/2021/9202127

**Published:** 2021-09-24

**Authors:** Yong Yang, Shuaishuai Zheng, Zhilu Ai, Mohammad Mahdi Molla Jafari

**Affiliations:** ^1^College of Food Science and Technology, Henan Agricultural University, Zhengzhou, Henan 450002, China; ^2^Key Laboratory of Staple Grain Processing, Ministry of Agriculture and Rural Affairs, Zhengzhou, Henan 450002, China; ^3^Department of Petroleum Engineering, Ahwaz, Faculty of Petroleum Engineering, Petroleum University of Technology (PUT), Ahwaz, Iran

## Abstract

This study is aimed at modeling biodigestion systems as a function of the most influencing parameters to generate two robust algorithms on the basis of the machine learning algorithms, including adaptive network-based fuzzy inference system (ANFIS) and least square support vector machine (LSSVM). The models are assessed utilizing multiple statistical analyses for the actual values and model outcomes. Results from the suggested models indicate their great capability of predicting biogas production from vegetable food, fruits, and wastes for a variety of ranges of input parameters. The values that are calculated for the mean relative error (MRE %) and mean squared error (MSE) were 29.318 and 0.0039 for ANFIS, and 2.951 and 0.0001 for LSSVM which shows that the latter model has a better ability to predict the target data. Finally, in order to have additional certainty, two analyses of outlier identification and sensitivity were performed on the input parameter data that proved the proposed model in this paper has higher reliability in assessing output values compared with the previous model.

## 1. Introduction

The main disposal pathways for FW (food waste) residues are treating via incineration or disposal in landfills. Given the very fast biodegradability of the food wastes in the presence of contaminating microorganisms, their disposal in landfills is very problematic [1, 2]. In addition, biodegradation within landfills necessitates a vast area, and greenhouse gases, e.g., methane, are generated with no profit gained via the energy produced through the biomass [3]. Thus, many nations have prohibited such a disposal option. From another viewpoint, given the high moisture content (>70%) of the organic matter, the incineration requires intensive amounts of energy with no energy recovery in some situations [4, 5]. Both options impose adverse impacts on human health and the environment [6, 7].

Therefore, since waste-to-energy techniques support reduced environmental impacts and partial replacement of fossil reserves, they are studied for disposal of organic wastes. One of the feasible approaches is AD (anaerobic digestion), which is an environmentally friendly technology for transforming liquid or solid organic wastes into biogas, which is convertible to beneficial energies (heat and/or electricity) [8, 9]. Anaerobic digestion is a complicated multistep biochemical degradation procedure conducted in the absence of O_2_, through which the microorganisms transform organic compounds into a gaseous mix mainly composed of CO_2_ (carbon dioxide), N_2_ (nitrogen), and CH_4_ (methane). Nonetheless, one can find other compounds in the composition, including H_2_ (hydrogen), H_2_S (hydrogen sulfide), O_2_ (oxygen), CO (carbon monoxide), and NH_3_ (ammonia). Also, trace amounts of siloxanes, dust particles, and halogenated and aromatic compounds are found in biogas; some of which can increase emissions, corrosion, and biohazards for human health. Biogas is also saturated with water. Also, this process generates sludge residues (or digests), which can be utilized as a fuel for energy generation subsequent to a drying treatment or directly for remediation of soil [10].

The conversion yield, the biogas composition, and the production rate are affected by the biomass nature, the configuration of biodigester, and the process characteristics [11, 12]. Given the diversity of handling and processing techniques, resources, local seasons and climates, and eating behaviors, the same kind of FWs may provide extremely variable features [13]. The amount of TSs (total solids) found in FW ranges from <2%w in liquid FWs to >90%w in solid FWs. The organic content (often ~90%) shown by the VS/TS ratio (in which the VS (volatile solids) represents the weight fraction convertible into gaseous materials) makes biomass a suitable candidate for anaerobic digestion. The C/N ratio varies between 3 and 55, paving the way for modifying the mixture C/N ratio to reach the optimum biodegradability for the food biomass. For most of the FWs, the acidity necessitates adding chemicals (e.g., alkali reagents) to stabilize biodigesters' pH. For each kind of FWs, the highest potential of methane production is within the 0.31–1.1 m^3^ CH_4_/kg range when volatile solids are added [14]. Thus, the electricity generated per 1 ton of fresh substances is within the 151.6-224.6 m^3^/t range for FVW and FW, which is nearly the same value acquired from chicken and cattle dungs (257.3 and 122.5 m^3^/t, respectively) [15].

As observed in [16–18] and others, the parameters contributing to biogas generation have been investigated. Nonetheless, only a few studies have simultaneously investigated more than a single factor. The simultaneous investigation of the interacting impact of a number of experimental scenarios presents invaluable data for optimization and prediction of the key features used in the experimental procedure of the entire studied scenarios. The problem is finding plausible standard experimental techniques for dataset compilation. Concerning optimization and prediction, the most routine methods pave the way for creating polynomial models correlating the response to the procedure irrespective of variables and their associated interactions [19]. Seman et al. concentrated on developing an association between parameters and evaluating the interactions between factors [20]. Therefore, the parameter optimization and response prediction of the process were founded on the basis of polynomial regression modeling (second-order model).

Another modeling instrument employed for better prediction is ANNs (artificial neural networks) [21]. The independent variables employed by Beltramo et al. in the artificial neural network model to assess the rate of biogas generation were TS, VFA, VS, acid detergent lignin, acid detergent fiber, ammonium nitrogen, neutral detergent fiber, OLR, and HRT [22]. The prediction error of the model was 6.24%, and the authors considered a coefficient of determination, *R*^2^ = 0.9, as the optimum result. By using an artificial neural network, Ghatak et al. optimized and modeled the prediction of particular biogas generation via the parameters including temperature, duration, and composition [23]. The neural model could anticipate the creation of biogas with an accuracy of 99.7%.

In this paper, we have tried to predict the values of biogas production from vegetables, fruits, and food wastes using two new models, ANFIS and LSSVM. First, a wide range of actual output data and input parameters affecting them were collected. Then, these two models were constructed and statistically evaluated, and compared. Finally, the results of these models were compared with the previously proposed models (in terms of accuracy), and the best model was proposed.

## 2. Description of Models

### 2.1. ANFIS

The adaptive network-based fuzzy inference system (ANFIS) algorithm is defined as a class of neural network techniques to address problems involving function approximation [24]. To put it in another way, an ANFIS structure is a combined information acquired from the fuzzy logic system and artificial neural network, and it consists of several membership function (MF) parameters optimized utilizing optimization algorithms [25]. Accordingly, the ANFIS structure, because of being particular, is significantly precise, and its reliance on real values is less than other machine learning algorithms, for instance, the artificial neural networks [25].

A typical ANFIS structure includes five layers, each of which has a number of nodes defined by their node functions [26]. Layers' association can be established using internal connections. The outputs of the previous layer are used as the inputs of the next layer. It is worth noting that the fuzzy inference system is utilized in the ANFIS technique as a fuzzy system. More specifically, for the inputs with two parameters, *x* and *y*, and output with a single parameter, *f*_*i*_, the rules governing an ANFIS structure are expressed as follows [27].

First rule: if *x* is *M*1 and *y* is *N*1 then *z* is *f*1(*x*, *y*)

First rule: if *x* is *M*2 and *y* is *N*2 then *z* is *f*2(*x*, *y*)

where fuzzy sets indicated by *M* and *N* and *f*_*i*_ (*x*, *y*) is representative of the first-order fuzzy inference system output.

The adaptive nodes included in the first layer are specified as follows:

(1) $$\begin{array}{c} O_{i}^{1}-\mu_{M_{i}}\left(x\right),\;\text{for }i=1,2, \end{array}$$

*O*_*i*_^1^ − *μ*_*M*_*i*−2__(*x*),  for *i* = 3, 4, where *μ*(*y*) and *μ*(*x*) indicate the membership functions.

Each node denoted by *π* is constant in the following layer.

(2) $$\begin{array}{c} O_{i}^{2}=W_{i}=\mu_{M_{i}}\left(x\right)\mu_{N_{i}}\left(y\right),\;\text{for }i=1,2, \end{array}$$

where *W*_*i*_ indicates the firing strength of the rule.

The third layer has constant nodes denoted by *N*. The corresponding node functions are applied to normalize the firing by dividing the *i*^th^ node's firing strength value by the all firing strength values' summation [28].

(3) $$\begin{array}{c} O_{i}^{3}=\frac{W_{i}}{\sum W_{i}},\;\text{for }i=1,2. \end{array}$$

The fourth layer includes adaptive nodes indicated by the square shapes.

(4) $$\begin{array}{c} O_{i}^{4}=\bar{W_{i}}f_{i}=\bar{W_{i}}\left(m_{i}X_{1}+n_{i}X_{2}+r_{i}\right),\;\text{for }i=1,2, \end{array}$$

where *f*_1_ and *f*_2_ indicate the fuzzy if-then rules defined as follows [29].

Rule 1: if *x* is *M*_1_ and *y* is *N*_1_ then *f*_1_=*p*_1_*x* + *q*_1_*y* + *r*_1_

Rule 1: if *x* is *M*_2_ and *y* is *N*_2_ then *f*_2_=*p*_2_*x* + *q*_2_*y* + *r*_2_

where *p*_*i*_, *q*_*i*_, and *r*_*i*_ indicate the consequential terms.

The overall output in the last layer is given by:

(5) $$\begin{array}{c} O_{i}^{5}=Y=\underset{i}{\sum }\bar{W_{i}}f_{i}=\bar{W_{1}}f_{1}+\bar{W_{2}}f_{2}=\frac{\sum W_{i}f_{i}}{\sum W_{i}}. \end{array}$$

Totally, the output is described as a linear combined consequential term [30].

### 2.2. LSSVM

The supervised least square support vector machine **(**LSSVM) algorithm developed in 1999 by Suykens et al. for solving problems stemmed from the regression together with function approximation. For the inputs denoted by *X*_*i*_ and the output denoted by *Y*_*i*_, the usual LSSVM nonlinear function is given as follows [31].

(6) $$\begin{array}{c} f\left(x\right)=\omega^{T}\phi \left(x\right)+b, \end{array}$$

where *f* indicates the connections between the target output and inputs, *w* denotes the *m*-dimensional weight vector, and *b* denotes the bias. The following equation is commonly used to solve the regression problems concerning the minimization theory [32]:

(7) $$\begin{array}{c} \min J\left(\omega ,e\right)=\frac{1}{2}\omega^{T}\omega +\frac{1}{2}\gamma \overset{N}{\underset{k=1}{\sum }}e_{k}^{2}. \end{array}$$

The following boundary conditions need to be considered:

(8) $$\begin{array}{c} y_{k}=\omega^{T}\phi \left(x_{k}\right)+b+e_{k}\;k=1,2,\cdots ,N, \end{array}$$

where *c* indicates the margin parameter and *e*_*k*_ indicates the error variable of *x*_*k*_. The LSSVM straightforward derivations lead to

(9) $$\begin{array}{c} f\left(x\right)=\overset{N}{\underset{k=1}{\sum }}a_{k}K\left(x,x_{k}\right)+b. \end{array}$$

The radial basis function is commonly used as a kernel function in regression faults due to its great efficiency, which is given by [33]

(10) $$\begin{array}{c} K\left(x,x_{k}\right)=e^{\left(-\left({\left\|x-x_{k}\right\|}^{2}/\sigma^{2}\right)\right)}. \end{array}$$

The *σ*^2^ in this equation indicates the squared bandwidth that needs to be estimated using optimization.

## 3. Materials and Methods

### 3.1. Sensitivity Analysis

In order to analyze the effects of individual inputs on the output value, a sensitivity analysis was carried out. Thus, the relevancy factor was decided as presented in the following to discover the effect of the individual inputs [34].

(11) $$\begin{array}{c} r=\frac{\sum_{i=1}^{n}\left(X_{k,i-}\bar{X_{k}}\right)\left(Y_{i}-\bar{Y}\right)}{\sqrt{\sum_{i=1}^{n}{\left(X_{k,i}-\bar{X_{k}}\right)}^{2}\sum_{i=1}^{n}{\left(Y_{i}-\bar{Y}\right)}^{2}}}. \end{array}$$

In this equation, ${\bar{X}}_{k}$ and *Y* stand for the average of the input, and the average of the *k*_th_ output, *Y*_*i*_ represents the *i*_th_ output, *N* represents the entire number of data points, and *X*_*k*,*i*_ stands for the *i*_th_ input value of the *k*_th_ parameter. Also, the *r* values range between -1 and 1. The less absolute value is interpreted as the fact that the input is less effective on the output parameter. In addition, the positivity or negativity of *r* is regarded as direct or reverse impacts of the concerned inputs; i.e., by increasing an input with negative *r* values, the target parameter is decreased; however, for the inputs with negative values of *r*, it is increased.

**Figure 1:**
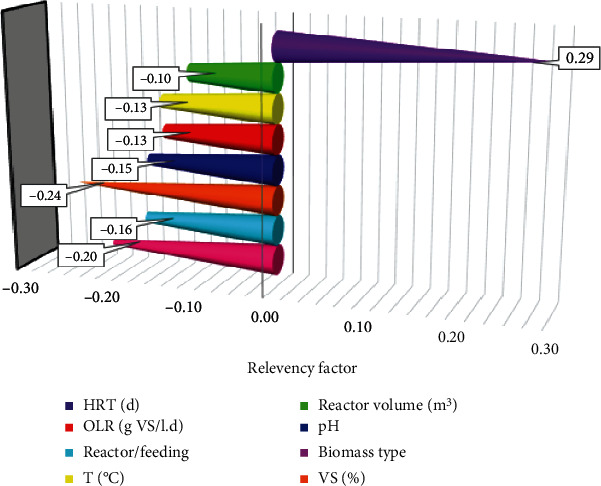
Sensitivity on various input parameters.

This research examined eight inputs that reflected a direct impact on the discussed target.  Figure 1 presents the analysis results, where the highest eigenvalue *n* is of the positive value of *r* = 0.29 that refers to HRT (d).

### 3.2. Data Gathering and Preanalysis Phase

In this phase, our study employed two different techniques to evaluate and predict the output parameters from the employed algorithms. Then, the data acquired in the experiments conducted in this research were employed for training the above-mentioned algorithms [35], whilst of the whole data points, about 25% of the same data points were used for the validation of those algorithms. Also, the dataset was subjected to the normalization procedure:

(12) $$\begin{array}{c} D_{k}=2\frac{x-x_{\min}}{x_{\max}-x_{\min}}-1. \end{array}$$

In the above equation, *D*_*k*_ represents the normalized value and *x* stands for the input value.

### 3.3. Identification of Outliers

Outlier or suspected data points featuring behaviors that differ from the major part of the databank show up in a large dataset typically. However, the same data points may affect model's accuracy and reliability. Hence, it seems necessary then to try to find such data in the proposed models, in particular for the training datasets. In case of neglecting some unrecognized impacts, some restrictions may be encountered in the model. In the other words, the analysis of outliers may provide us with an insight on the same restrictions, which are the benefits of the discussed analysis. In order to eliminate the outlier data, the leverage technique was used, which requires determining the deviation of the predictive tool from the concerned real data [34, 36]. The deviation which is also termed as standardized crossvalidated residuals creates a Hat matrix, which can be determined on the basis of the equation below in this study:

(13) $$\begin{array}{c} H=X{\left(X^{t}X\right)}^{-1}X^{t}, \end{array}$$

where *X* stands for an *N* × *P* matrix. *N* and *P*, respectively, represent the entire number of data points and the input parameters. *T* and -1 are called transpose and inverse operators, respectively. In addition, the equation below was used to explicate a warning leverage value:

(14) $$\begin{array}{c} H^{*}=\frac{3n}{\left(p+1\right)}. \end{array}$$

**Figure 2:**
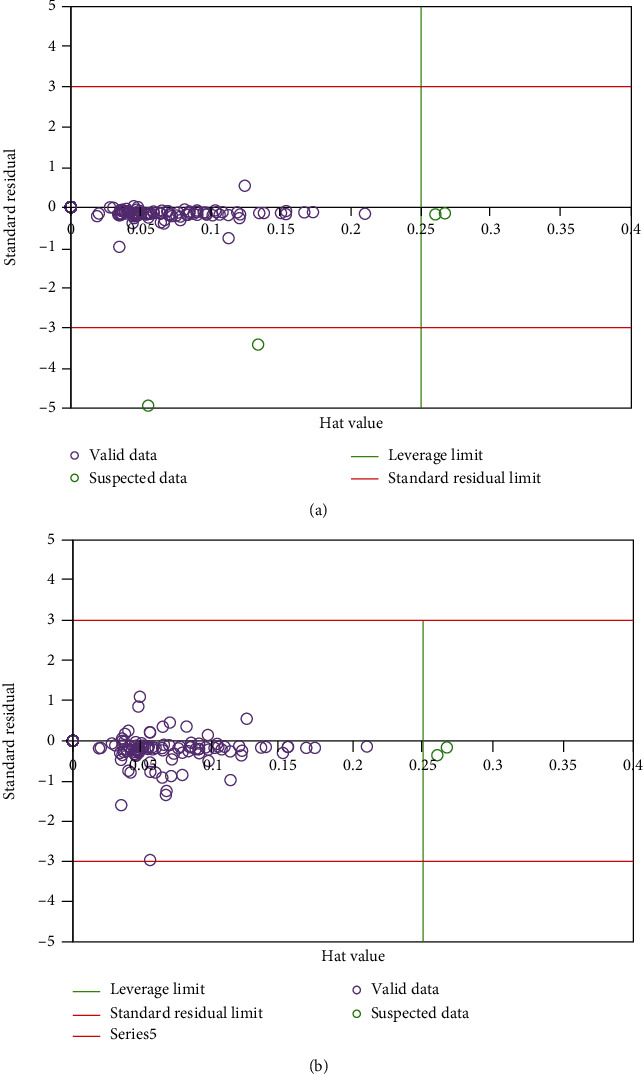
Analysis to identify outlier's data in models (a) LSSVM and (b) ANFIS.

The practical region is delineated within 0 ≤ *H* ≤ *H*∗ and *R* = ±3 rectangular area. According to the red points observed in Figure 2, only a number of 20 suspected data were discovered amongst the entire dataset.

## 4. Results and Discussion

**Figure 3:**
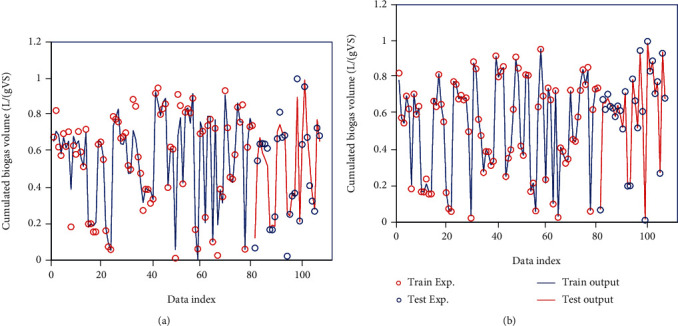
Simultaneous viewing of real and simulated output data using models (a) LSSVM and (b) ANFIS.

The two computational techniques developed in this work are ANFIS and LSSVM used to estimate the target values. Upon splitting the dataset into the testing and training datasets, of the whole data points, 75% were employed to make use of the above-mentioned model for determining the outputs. Then, the training process performance is expressible through a comparison made between the real values and the predicted ones in this step. Alternatively, the comparison made in the testing phase presents a better idea about the model's accuracy in unclear circumstances, which is called model generalization.  Figure 3 presents the simultaneous comparison of the experimental and determined targets for the whole models trained in testing and training databanks. Also, as Figure 3 suggests, the calculated constants may include the experimental points featuring plausible performances.

**Figure 4:**
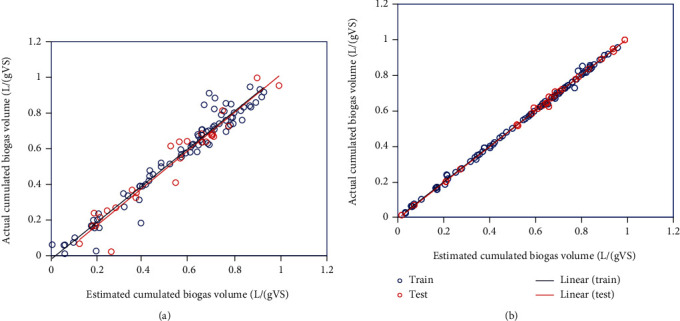
Linear regression diagrams to determine the accuracy of models (a) LSSVM and (b) ANFIS.

Thus, it is evident that the proposed LSSVM model can estimate outputs with higher accuracy. In order to evaluate the usability of the proposed models, various mathematical and graphical techniques were used. In Figure 4, the crossplots or regression are presented exhibiting the capability of the suggested algorithms in estimating the target values. It is clear that the data points are highly concentrated around the bisector line.

**Figure 5:**
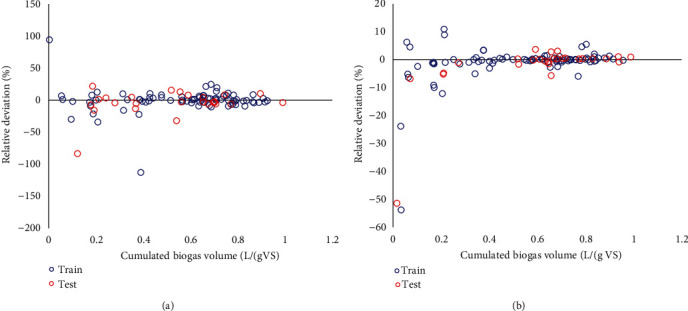
The deviation plots for the (a) LSSVM and (b) ANFIS models.

Figure 5 presents the deviation plots for the ANFIS and LSSVM models and shows the outputs vs. relative deviation for testing and training steps. Most of the determined deviations are found in the vicinity of the zero error line. In addition, the deviations compaction in the LSSVM model is clearer than the other model. The same compaction reflects the accuracy of prediction for this model.

Table 1 reports the mathematical indexes determined for the presented models. The higher values of *R*^2^, and also, the lower values of RMSE, MRE, STD, and MSE are observable for the proposed models, reflecting their good capability in estimating the output values.

Also, the models of ANFIS and LSSVM and the rest of the techniques found in the literature to decide the target values, e.g., those presented by the authors such as Neto and colleagues were compared. In 2021, they used the artificial neural network method to predict this parameter [35]. Compared to other models, the LSSVM model with *R*^2^ = 0.998 features the most optimal performance. The same comparison reveals that the minimum accuracy is attributable to the ANN model with *R*^2^ = 0.6167.

## 5. Conclusions

In this study, two accurate techniques, i.e., ANFIS and LSSVM, were presented successfully to estimate biogas production. The developed instruments used for estimation may help the scholars in suggesting a new efficient measurement technique. According to the statistical analyses, the LSSVM model can lead to the most accurate results with the best values of STD, RMSE, *R*^2^, MSE, and MRE. Given the above results, compared to the rest of the computational techniques, the LSSVM model presented a superb performance in terms of validity, accuracy, and generalization. Additionally, a sensitivity analysis was conducted in order to reflect the effect of input parameters on the target values which showed that HRT (d) has the greatest effect on the output parameter.

## Figures and Tables

**Table 1:** Results of various statistical analyzes to determine the accuracy of the two models ANFIS and LSSVM in predicting real values.

Model	Phase	*R* ^2^	MRE (%)	MSE	RMSE	STD
LSSVM	Train	0.998	2.762	0.0001	0.0113	0.0091
	Test	0.998	3.521	0.0001	0.0111	0.0082
	Total	0.998	2.951	0.0001	0.0111	0.0089
ANFIS	Train	0.949	22.070	0.0036	0.0598	0.0464
	Test	0.936	51.064	0.0047	0.0683	0.0494
	Total	0.946	29.318	0.0039	0.0683	0.0471

## Data Availability

The data used to support the findings of this study are provided within the article.
